# Epidemiological features of brucellosis and factors affecting its treatment failure and relapse in Qom Province, Iran

**DOI:** 10.4314/gmj.v55i3.5

**Published:** 2021-09

**Authors:** Abolfazl Mohammadbeigi, Abedin Saghafipour, Amir Hamta, Salman Khazaei, Atefeh Maghsoudi, Saeed Shams

**Affiliations:** 1 Neuroscience Research Center, Department of Epidemiology and Biostatistics, Qom University of Medical Sciences, Qom, Iran; 2 Department of Public Health, Faculty of Health, Qom University of Medical Sciences, Qom, Iran; 3 Department of Social Medicine, Faculty of Medical Sciences, Qom University of Medical Sciences, Qom, Iran; 4 Research Center for Health Sciences, Hamadan University of Medical Sciences, Hamadan, Iran; 5 Student Research Committee, Qom University of Medical Sciences, Qom, Iran; 6 Cellular and Molecular Research Center, Qom University of Medical Sciences. Qom, Iran

**Keywords:** Brucellosis, Relapse, Risk factors, Treatment failure, Iran

## Abstract

**Background:**

Brucellosis is one of the major health problems in many areas of the world, especially in the Mediterranean and the Middle East regions.

**Objective:**

To determine the epidemiological characteristics, clinical signs, and risk factors of relapse rate in patients with brucellosis, Qom Province, Iran.

**Methods:**

A descriptive-analytic study was conducted on 410 confirmed brucellosis cases in Qom Province, central Iran, from 2015 to 2019, based on epidemiological checklists and according to the Iran Ministry of Health and Medical Education (MOHME). Univariate and multiple logistic regression analyses were conducted using Stata software version 14.

**Results:**

The relapse rate of brucellosis was 6.6% until nine months after starting the treatment, and all recurrent cases were infected by *Brucella melitensis*. Based on univariate logistic regression analysis, the delayed treatment and type species of *Brucella* were significant factors affecting the relapse of brucellosis. The relapse rates were 5.4%, 6.2%, and 20.0% in patients whose delayed treatments were <50, 51–150, and >151days, respectively. Based on the multiple logistic regression, it was observed that delayed treatment >50 days increased the rate of relapse more than four times.

**Conclusion:**

The delayed initiation of treatment was a significant factor influencing the relapse of brucellosis; therefore, it is necessary to provide enough diagnostic and laboratory facilities, and people need to be educated about the signs and symptoms of the disease.

**Funding:**

Funding for this research was provided by the Research and Technology Center of Qom University of Medical Sciences, Qom, Iran

## Introduction

Brucellosis is a serious public health issue that may lead to economic problems in many areas of the world, especially in the Mediterranean and the Middle East regions.^1^ The World Health Organization (WHO) estimates that the number of new brucellosis cases in the world is more than 500,000 per year.^2^ Although brucellosis has been controlled in many developed countries, it remains an important health problem in developing countries.^3^ The disease is caused by *Brucella* spp. Those are gram-negative, facultative coccobacilli, small, non-motile, and nonspore-forming.^4^ Brucellosis in humans is mainly caused by the consumption of raw milk and its products and undercooked meat. Other ways of transmitting the disease include the entry of bacteria through the membranes, skin, inhalation, droplets, and dust particles containing bacteria. Farmers, laboratory technicians, slaughterhouse workers, and veterinarians are more at risk of the disease^5^ The disease is reported in all regions of the world, especially in the countries around the Mediterranean Sea (Southern Europe, northern and Eastern Africa), the Middle East, India, and Central Asia.^1^

The disease has been endemic in Iran, especially in Qom Province, with a prevalence of 0–10 per 100,000 people since many years ago.^6^ The incubation period of brucellosis is different, from one to several months (from four weeks to several months) classified as acute, sub-acute, chronic, localized, and relapsing forms in humans.^7^ The symptoms of acute illness are fever, chills, headache, muscular and joint pains, weakness, fatigue, agitation, night sweats, and loss of appetite. Brucellosis is a multisystemic disease that may affect various organs, including digestion, cardiovascular, hematopoietic, neurological, skeletal, pulmonary, skin, and eye.^8^ Illness recurrence occurs after three to six months of the treatment that may be associated with relapse.^9^ If the disease symptoms remain for more than a year, it is called chronic brucellosis. This status is characterized by the persistence of infectious agents in different tissues such as bones, joints, liver, spleen, and kidneys, which can be specified using a high titer of IgG that persists for a long time in the serum. The chronic form of the disease should be distinguished from non-specific complaints such as fatigue in patients or recovery from the recovery phase. In these conditions, fever and high levels of IgG are not observed.^10^ It is estimated that about 10% of cases of brucellosis relapse after antibiotic treatment. This may be due to the intracellular nature of the bacteria and the absence of exposure to prescribed antibiotics and host defence mechanisms.^11^ Therefore, this study aimed to determine the epidemiological characteristics, clinical signs, risk factors of relapse rate and treatment failure in patients with brucellosis in Qom Province, central Iran, from 2015 to 2019.

## Methods

This study was a descriptive-analytic study based on the registered data of all human brucellosis cases diagnosed in governmental and private clinics of all five districts in Qom Province, central Iran, in 2015–2019. In this study, the brucellosis-cases data were reported to the department of prevention and diseases control in provincial health centres from all comprehensive health centres, provincial reference laboratories (PRL), and private clinics by public health experts who visited these centres monthly in the form of communicable disease reporting and surveillance system. It should be mentioned that these data were collected by completing the standard data forms approved by the CDC of Iran MOHME, including demographic, epidemiological (relapse and failure of treatment), clinical, and laboratory (microbial causative agents of the disease) information, and all districts of the province were considered as the study areas. All patients with brucellosis in five years (2015–2019) were included in the study. Patients' information was collected through laboratories, private physician offices, records in hospitals and health centres in Qom province.

The epidemiological checklists were completed according to the standards of Iran MOHME (the Center for Diseases Management and Zoonotic Disease) by the health staff, monthly transferred to the provincial health centre. The classification definition of brucellosis cases included the following: i) all individuals who had clinical signs consistent with brucellosis associated with an epidemiological link with suspected or definite cases of animal brucellosis or contaminated animal products (suspected case), ii) the suspected cases diagnosed with the Wright test having a headline equal to or greater than 1:80 (probable case), and iii) suspected or probable cases associated with a definitive laboratory diagnostic index (definite case). The definitive laboratory diagnostic index includes a) detection of the disease agent from clinical specimens using the culture media, b) 2ME≥ 1:40, and c) Coombs-Wright test with three dilutions greater than the Wright test.

All patients' information was confidential and included in the code without a name, and ethical principles were considered in the analysis and report of results. In addition, ethical approval was obtained from the ethical committee of Qom University of Medical Sciences, Qom, Iran (IR.MUQ.REC.1398.140). The study included all the eligible confirmed patients based on the WHO classification of brucellosis in Qom Province. Data were presented as mean ± standard deviation for continuous variables and frequency/percentage for categorical variables.

By considering relapse as a response variable, this study was carried out in two stages: For model building, first, univariate analysis was utilized by univariate logistic regression to distinguish potential confounders (reported in table 2 and 3) in order not to miss any important ones; the significant level was assigned at 0.2. In the next stage, multiple logistic regression was done on all variables that were significant in univariate analysis to calculate the adjusted odds ratio. In this stage, the significant level was assigned at 0.05. The statistical analyses were run by Stata software version 14.

**Table 2:** Logistic regression analyses results of potential risk factors for relapse of patients with brucellosis in Qom Province during 2015–2019

Risk factors		Total number (%) of cases	Relapse N (%)	Non-relapse N (%)	Unadjusted Odd Ratio (95% CI)	P- value	Adjusted Odd Ratio (95% CI)	P- value
**Gender**	Male	266 (64.9)	13 (4.9)	253 (95.1)	1		1	
	Female	144 (35.1)	14(9.7)	130 (90.3)	**2.09 (1.16,4.59)**	**0.06**	1.42 (0.61,3.28)	0.260
**Age group**	< 15	32 (7.8)	1(3.1)	31(96.9)	1		-	-
	15–45	210 (51.2)	12(5.7)	198 (94.3)	0.35 (0.04,2.79)	-	-	-
	> 45	168 (41.0)	14(8.3)	154 (91.7)	0.67 (0.30,1.48)	-	-	
**Residency**	Urban	291 (71.0)	20(6.9)	271 (93.1)	1		-	-
	Rural	119 (29.0)	7(5.9)	112 (94.1)	0.85 (0.35,2.60)	-	-	-
**Nationality** 1	Iranian	396 (96.6)	27(6.8)	396 (93.2)	1		-	-
	Non-Iranian	14 (3.4)	0 (0)	14(100)	<0.001(<0.01, NA)	-	-	-
**Delayed treatment**	<50	278 (70.6)	15 (5.4)	263 (94.6)	1		1	
	51–150	81 (20.6)	5 (6.2)	76(93.8)	4.35 (1.49,11.11)	0.003	4.35(1.54,8.97)	0.005
	>150	35 (8.9)	7 (20)	28 (80)	3.84(1.11,7.87)	0.031	4.17(1.15,8.17)	0.03
**contact with live animals**	Yes	142 (34.6)	11 (7.7)	131 (92.3)	1.32 (0.60,2.95)	0.49	-	-
	No	268 (65.4)	16 (6)	252 (94)	1		-	-
**Other types of contact** *	Yes	120 (29.3)	5 (4.2)	115 (95.8)	0.53 (0.2,1.43)	0.21	-	-
	No	290 (70.7)	22 (7.6)	268 (92.4)	1			-
**History contact with** **livestock**	Yes	225 (57)	14 (6.2)	211 (93.8)	0.8 (0.37,1.76)	0.58	-	-
	No	170 (43)	13 (7.6)	157 (92.4)	1		-	-
**Keeping of livestock**	Yes	84 (20.5)	4 (4.8)	80 (95.2)	0.61 (0.21,1.79)	0.36	-	-
	No	326 (79.5)	25 (7.7)	301 (92.3)	1		-	-
**History of brucellosis** **in family members**	Yes	59 (14.7)	7 (11.9)	52 (88.1)	1.97 (0.8,4.77)	0.51	-	-
	No	343 (85.3)	22 (6.4)	321 (93.6)	1		-	-
**Brucella species**	*B. melitensis*	391(95.4)	29 (7.4)	362 (92.6)	<0.001(<0.01, NA)	>0.99	-	-
	*B. Abortus*	19 (4.6)	0 (0)	19 (100)	Reference		-	-

* Slaughtering of livestock and contact with blood, and mucous membranes

1 Because of low incidence statistical analysis is not feasible.

**Table 3:** Symptoms and signs predicting relapse in patients with brucellosis in Qom Province during 2015–2019

Symptoms and signs			Total cases (%)	Relapse n(%)	No relapse n(%)	Unadjusted Odd Ratio (95% CI)	P- value	Adjusted Odd Ratio (95% CI)	P- value
**Symptoms**	Fever	Yes	292 (71.2)	15 (5.1)	277 (94.9)	0.48 (0.22,1.06)	0.07	0.43 (0.19, 0.1)	0.04
		No	118 (28.8)	12 (10.2)	106 (89.8)	1		1	
	Joint and back pain^1^	Yes	264 (64.4)	16 (6.1)	248 (93.9)	0.8 (0.36,1.76)	0.56	-	-
		No	146 (35.6)	11 (7.5)	135 (92.5)	1		-	-
	Weight loss^1^	Yes	162 (39.5)	8 (4.9)	154(95.1)	0.63 (0.27,1.48)	0.28	-	-
		No	248 (60.5)	19 (7.7)	229 (92.3)	1			-
	Anorexia^1^	Yes	207 (50.5)	13 (6.3)	194 (93.7)	0.91 (0.42,1.97)	0.80	-	-
		No	203 (49.5)	14 (6.9)	189 (93.1)	1		-	-
	weakness	Yes	94 (22.5)	10 (10.6)	84 (89.4)	2.09 (1.03,4.77)	0.08	2.5 (1.05,5.89)	0.04
		No	316 (77.1)	17 (5.4)	299 (94.6)	1		1	
**Signs**	Hepatosplenomegaly^1^	Yes	9 (2.2)	1 (11.1)	8(88.9)	1.82 (0.22,14.29)	0.58	-	-
		No	401 (97.8)	26 (6.5)	375 (93.5)	1			-
	Arthritis^1^	Yes	353 (86.1)	23 (6.5)	330 (93.5)	0.93 (0.31,2.78)	0.88	-	-
		No	57 (13.9)	4 (7)	53 (93)	1			
**Antibiotic Treatment regimen**	Gentamicin^1^	Yes	141 (34.4)	7 (5)	134 (95)	0.65 (0.27,1.59)	0.34	-	-
		No	269 (65.6)	20 (7.4)	249 (92.6)	1			-
	Rifampin	Yes	362 (88.3)	24 (6.6)	338 (93.4)	1.07 (0.31,3.71)	0.92	-	-
		No	48 (11.7)	3 (6.3)	45 (93.8)	1		-	-
	Co-trimoxazole^1^	Yes	70 (17.1)	5 (7.1)	65 (92.9)	1.12 (0.41,3.04)	0.84	-	-
		No	340 (82.9)	22 (6.5)	318 (93.5)	1		-	-
	Streptomycin	Yes	72 (17.6)	2 (7.4)	70 (18.3)	0.36 (0.49,1.54)	0.17	0.42 (0.56,1.86)	0.25
		No	338 (82.4)	25 (7.4)	313 (92.6)	1		1	
	Doxycycline^1^	Yes	354(86.3)	23 (85.2)	331 (86.4)	0.91 (0.31,2.71)	0.86	-	-
		No	56 (13.7)	4 (7.1)	52 (92.9)	1		-	-
	Tetracycline^1^	Yes	10 (2.4)	1 (10)	9 (90)	1.59 (0.2,12.5)	0.63	-	-
		No	400 (97.6)	27 (6.6)	383 (93.4)	1		-	-

1 Significant level for including in adjusted model was considered 0.2 and variables that were not significant in unadjusted form were excluded.

## Results

During the study period from March 2015 to March 2019, 410 confirmed cases of brucellosis were diagnosed in Qom Province who were all analyzed in this study. The mean age of patients was 39.87± 19.93 years ranging from 1 to 86 years old. Of all the patients, 266 cases (64.9%) were male, and 144 cases (35.1%) were female. Therefore, the sex ratio of brucellosis was calculated as 1.85. According to Table 1, the residency area of 291 patients (71.0%) was urban and 119 patients (29.0%) rural. The majority of patients (168 cases, 41.0%) were >40 years old, and 84.3% of patients had lower-diploma educations. Clinical data showed that until nine months after starting the treatment in all 410 patients, the recurrence of brucellosis occurred in 27 cases (6.6%) as the number of serologic tests increased and all recurrent cases were affected by *Brucella melitensis*. Table 1 shows that 396 patients (96.6%) were Iranian, and 391 cases (95.4%) were affected by *B. melitensis*. History of contact with livestock was reported in 225 patients (57.0%), and livestock keeping and contact with live animals were also reported in 84 patients (20.5%) and 142 patients (34.6%), respectively.

**Table 1:** Demographic and epidemiological characteristics of patients with brucellosis in Qom Province during 2015–2019

Variable		N (%)
**Gender**	Male	266 (64.9)
	Female	144 (35.1)
**Age groups**	< 15	32 (7.8)
	15–45	210 (51.2)
	> 45	168 (41.0)
**Residence place**	Urban	291 (71.0)
	Rural	119 (29.0)
**Nationality**	Iranian	396 (96.6)
	Non-Iranian	14 (3.4)
**Delayed treatment**	<50	278 (70.6)
	51–150	81 (20.6)
	>150	35 (8.9)
**Occupational contacts** **(contact with live animals)**	Yes	142 (34.6)
	No	268 (65.4)
**Slaughtering of livestock** **and contact with blood and** **mucous membranes**	Yes	120 (29.3)
	No	290 (70.7)
**History of contact with** **livestock**	Yes	225 (57.0)
	No	170 (43.0)
**Keeping of livestock**	Yes	84 (20.5)
	No	326 (79.5)
**History of brucellosis in** **household family members**	Yes	59 (14.7)
	No	343 (85.3)
***Brucella* species**	*B. melitensis*	391(95.4)
	*B. abortus*	19 (4.6)

**Figure 1:**
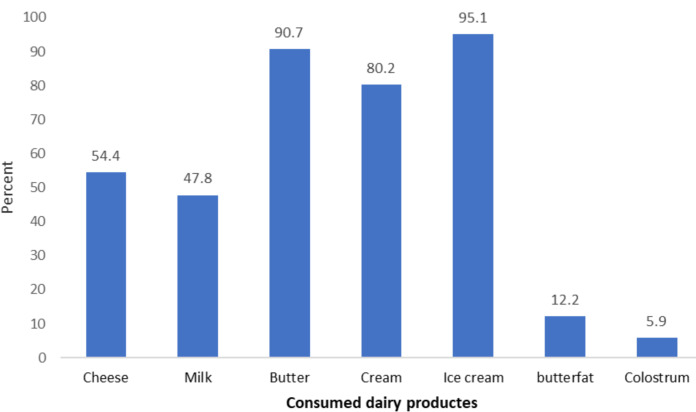
Types of consumed unpasteurized dairy products by brucellosis cases in Qom province during 2015–2019

As observed in Figure 1, among those infected with brucellosis, 95.1%, 90.7%, and 80.2% consumed ice cream, butter, and cream, respectively. Moreover, the results presented in Table 2 show the unadjusted and adjusted odds ratio of the demographic-related factors of relapse. Based on univariate analysis, the delayed treatment and type of *Brucella* were significant factors related to the relapse of brucellosis.

However, sex, age group, residency area, nationality, occupational contact with live animals, slaughtering livestock and contact with blood, and mucous membranes, history of contact with livestock, keeping livestock, and history of brucellosis in household family members did not show any significant relationships (p>0.05).

However, based on multiple logistic regression, the delayed treatment was the most important factor of relapse, and increasing the delay raised the risk of relapse. The relapse rates were measured as 5.4%, 6.2%, and 20.0% in patients with delayed treatments as <50, 51–150, and >151days, respectively. The univariate regression model showed that gender and delayed treatment were significant factors of brucellosis relapse. According to Table 2, female sex was a factor of relapse (OR=2.09, 95% CI; 1.16, 4.59). Moreover, delay in brucellosis treatment from 50–150 days could increase the relapse rate (OR= 4.35; 95% CI; 1.49, 11.11), and the delayed treatment of more than 150 days could increase the relapse rate up to OR=3.84; 95% CI: 1.11, 7.87). Additionally, based on multiple logistic regression, gender was not a significant factor. Nevertheless, delayed treatment was the most important factor of relapse. Based on the multiple logistic regression, the delayed treatment >50 days increased relapse rate more than four times.

As shown in Table 3, fever and weakness were the most important signs and symptoms as predictors of relapse in patients (p<0.05). Multiple logistic regression revealed that the absence of fever was related to a higher relapse in patients and could increase the rate of relapse (OR=0.43 (95% CI: 0.19, 0.10), p=0.04). In addition, weakness was a sign that increased the chance of relapse. The rate of relapse was estimated to be 10.6% in patients with weakness and 5.4% in patients without weakness. Therefore, based on the adjusted regression analysis, lack of weakness was related to lower relapse rates (OR=2.5(1.05, 5.89), p=0.04). Moreover, no significant relationship was found between the type of medication and relapse occurrence (p>0.05). Table 4 demonstrates no significant relationship between relapse and each of the consumed unpasteurized dairy products (p>0.05).

**Table 4:** The relationship between types of consumed unpasteurized dairy products and relapse among brucellosis cases from 2015 to 2019

Type of unpasteurized dairy products		Relapse N (%)	Non relapse N (%)	Unadjusted Odd Ratio (95% CI)	P- value	Adjusted Odd Ratio (95% CI)	P- value
**Cheese**	Yes	15 (6.7)	208 (93.3)	1.32 (0.6, 2.86)	0.50	-	-
	No	14 (7.5)	173 (92.5)	Reference			
**Milk**	Yes	9(4.6)	187 (95.4)	0.53 (0.23, 1.2)	0.119	0.71 (0.31,1.64)	0.41
	No	20 (9.3)	194 (90.7)	Reference			
**Butter**	Yes	28(7.5)	344 (92.5)	0.36 (0.93, 2.71)	0.32	-	-
	No	1 (2.6)	37 (97.4)	Reference			
**Cream**	Yes	22 (6.7)	307 (93.3)	1.48 (0.6,3.58)	0.41	-	-
	No	7 (8.6)	74 (91.4)	Reference			
**Ice cream**	Yes	28 (7.2)	362 (92.8)	0.74 (1.86,5.89)	0.77	-	-
	No	1 (5)	19 (95)	Reference			
**butterfat**	Yes	4 (8)	46 (92)	1.29 (0.43, 3.85)	0.67	-	-
	No	25 (6.9)	335 (93.1)	Reference			
**Colostrum**	Yes	1 (4.2)	23 (95.8)	0.56 (0.27, 4.35)	0.57	-	-
	No	28 (7.3)	358 (92.7)	Reference			

## Discussion

This study assessed the profile of 410 brucellosis cases in Qom Province in Iran, and the results showed that most cases were males, urban dwellers, and economically active. Having contacts with livestock and consumption of ice cream, butter, and cream were found to be the common ways of transmission. The rate of relapse was considerable (6.6% of cases), and the delayed initiation of treatment was a significant factor affecting the relapse of brucellosis. The lack of fever and weakness were the most important signs and symptoms in relapse cases, which was another important clinical finding of the current study. Along with evidence demonstrating the higher risk of brucellosis in males,^12^–^14^, males can be more susceptible to the disease. However, a study conducted in Hamadan Province has been reported that females were more susceptible to the disease.^15^ This contradiction may be because in societies where animal husbandry is prevalent, the rate of infection in women is also high. The results also revealed that the proportion of brucellosis cases was higher in productive ages. This finding agrees with the findings of previous studies.^15^,^16^ It is usually the case that because of their occupation requirements, people of this age group are more likely to be in contact with livestock.

Despite the results of several studies reporting the abundance of brucellosis patients in rural areas,^14^,^17^ in this study, the frequency of brucellosis in urban areas was twice more than that of the rural population. Nearly 70% of Qom population are urban dwellers, and obviously, the frequency of the disease is even higher in the urban population. Despite the use of standard therapeutic drugs for brucellosis,^18^ per cent of patients face treatment failure and relapse.^19^

In line with the findings of this study, the results of a study carried out in Hamadan Province indicated that the rate of relapse among brucellosis patients was 6.4%.^20^ Yet, in another study conducted by Alavi et al. in Ahvaz,^19^ 18.3% of cases had relapsed. This discrepancy may be due to differences in the years of studies as the study by Alavi et al. 19 was carried out on identified patients in 2004–2006 and progress in therapeutics methods in recent years may justify this difference. Although risk factors for infection are well-known, risk factors for relapse are not yet clearly understood, and evidence in this regard is scarce. Generally, some factors like the type of *Brucella* species, immunity suppression, and delay in initiation of treatment are associated with the relapse of *Brucella*.^21^ Hasanjani Roushan et al. found that treatment regimen is the predictor of relapse for brucellosis cases.^22^

In the current study, delay in treatment was a risk factor for the acquisition of relapse. Consistent with the findings of this research, Alavi et al. observed that many patients with relapse had a long duration of time between the appearance of symptoms and onset of treatment.^19^ The findings of this study showed that the rate of fever was milder in relapse cases. Clinical signs are usually milder and less exclusive in relapse. However, the present study had several limitations. First, the registrybased data were used, and the validity of the data depends on the quality of data collection and registration. Second, the low occurrence of relapse caused sparse data in some cells of tables and consequently unstable estimations. Therefore, it is recommended that due to the lack of valid and reliable data regarding the epidemiology of relapse of brucellosis in Iran, longitudinal studies with larger sample sizes are needed to understand the predictors of relapse in brucellosis cases and control the disease more efficiently.

## Conclusion

The study results demonstrated that in Qom Province, the males, urban dwellers, and 15–45 -years old are more prone to brucellosis. The delayed initiation of the treatment is a significant factor related to the relapse of brucellosis; therefore, it is necessary to provide enough diagnostic and laboratory facilities on the one hand and try to educate people about the signs and symptoms of the disease on the other.

## References

[1] Musallam II, Abo-Shehada MN, Hegazy YM, Holt HR, Guitian FJ (2016). Systematic review of brucellosis in the Middle East: disease frequency in ruminants and humans and risk factors for human infection. Epidemiol Infect.

[2] World Health Organization Regional Office for South-East Asia (WHO/SEARO) (2014). A brief guide to emerging infectious diseases and zoonoses.

[3] Franc KA, Krecek RC, Hasler BN, Arenas-Gamboa AM (2018). Brucellosis remains a neglected disease in the developing world: a call for interdisciplinary action. BMC Public Health.

[4] Olsen SC, Palmer MV (2014). Advancement of knowledge of Brucella over the past 50 years. Vet Pathol.

[5] Hasanjani Roushan MR, Ebrahimpour S (2015). Human brucellosis: An overview. Caspian J Intern Med.

[6] Golshani M, Buozari S (2017). A Review of Brucellosis in Iran: Epidemiology, Risk Factors, Diagnosis, Control, and Prevention. Iran Biomed J.

[7] Khan MZ, Zahoor M (2018). An Overview of Brucellosis in Cattle and Humans, and its Serological and Molecular Diagnosis in Control Strategies. Trop Med Infect Dis.

[8] Hasanjani Roushan MR, Ebrahimpour S, Moulana Z (2016). Different Clinical Presentations of Brucellosis. Jundishapur J Microbiol.

[9] Hasanjani Roushan MR, Moulana Z, Mohseni Afshar Z, Ebrahimpour S (2015). Risk Factors for Relapse of Human Brucellosis. Glob J Health Sci.

[10] Hajia M, Keramat F (2003). Study On the rate of Brucellosis relapse efficiency Different Treatment Protocols in among Hospitalized Patient in Educational Hospital of Hamadan. J Mil Med.

[11] Meng F, Pan X, Tong W (2018). Rifampicin versus streptomycin for brucellosis treatment in humans: A metaanalysis of randomized controlled trials. PLoS One.

[12] Eini P, Keramat F, Hasanzadehhoseinabadi M (2012). Epidemiologic, Clinical and Laboratory Findings of Patients with Brucellosis in Hamadan, West of Iran. J Res Health Sci.

[13] Jia P, Joyner A (2015). Human brucellosis occurrences in inner mongolia, China: a spatio-temporal distribution and ecological niche modeling approach. BMC Infec Dis.

[14] Sofian M, Aghakhani A, Velayati AA, Banifazl M, Eslamifar A, Ramezani A (2008). Risk factors for human brucellosis in Iran: a case-control study. Int J Infec Dis.

[15] Khazaei S, Karami M, Mohammadbeigi A, Ayubi E, Shojaeian M, Mansouri K (2018). Spatio-Temporal analysis of brucellosis in Hamadan Province, West of Iran: 2009–2015. Adv Hum Biol.

[16] Zhang WY, Guo WD, Sun SH, Jiang JF, Sun HL, Li SL (2010). Human brucellosis, Inner Mongolia, China. Emerg Infect Dis.

[17] Hussini AS, Ramlawi AM (2004). Brucellosis in the West Bank, Palestine. Saudi Med J.

[18] de Gonzalez AB, Cox DR (2007). Interpretation of interaction: A review. The Annals of Applied Statistics.

[19] Alavi M, Alavi MR, Alavi L, Relapsed Human (2009). Brucellosis and related risk factors. Pak J Med Sci.

[20] Nematollahi S, Ayubi E, Karami M, Khazaei S, Shojaeian M, Zamani R (2017). Epidemiological characteristics of human brucellosis in Hamadan Province during 2009-2015: results from the National Notifiable Diseases Surveillance System. Inter J Infec Dis.

[21] Young EJ, Mandel GI, Bonnet JE, Dolin R (2005). Brucella species. Principle and practice of infectious disease.

[22] Roushan MRH, Moulana Z, Afshar ZM, Ebrahimpour S (2016). Risk factors for relapse of human brucellosis. Glob J Health Sci.

